# Perspectives from Underrepresented Patients and Families Following Biomarker‐Informed ADRD Diagnoses: Insights from the PARADE Study

**DOI:** 10.1002/alz.090816

**Published:** 2025-01-09

**Authors:** Melissa L Knox, Megan G. Witbracht, Joshua D Grill, Jennifer H Lingler

**Affiliations:** ^1^ University of Pittsburgh, Pittsburgh, PA USA; ^2^ University of Pittsburgh Alzheimer’s Disease Research Center (ADRC), Pittsburgh, PA USA; ^3^ Institute for Memory Impairments and Neurological Disorders, University of California, Irvine, Irvine, CA USA; ^4^ University of California, Irvine, Irvine, CA USA

## Abstract

**Background:**

As the landscape of ADRD diagnoses evolves to include biomarker testing, there is a pressing need to understand the unique experiences, challenges, and support needs of families undergoing evaluations of cognitive decline, particularly in a manner that prioritizes cultural considerations from voices historically underrepresented in ADRD research. The current study aims to understand the AD biomarker disclosure journey of persons from underrepresented groups with the goal of informing culturally responsive approaches to the care of patients and their families navigating the complexities of ADRD diagnoses.

**Method:**

Virtual focus groups are being conducted over a secure video conferencing platform, with a trained facilitator guiding the discussion. Participants represent a purposively selected subsample of those enrolled in Patient And family member Reactions to biomarker‐informed ADRD DiagnosEs (PARADE NIH RF1AG080591), a current add‐on to the New IDEAS study, a CMS coverage with evidence development study of amyloid PET with an explicit objective to represent diverse patients by enrolling >50% who identify as Black, African American, or Hispanic. Employing a semi‐structured interview approach, the focus group guide consists of 5 lead questions and follow‐up probes, aiming to explore participants' experiences in receiving amyloid PET results and to characterize their post‐disclosure information and support needs. Verbatim transcripts of focus group recordings will be analyzed using thematic analyses.

**Result:**

Preliminary findings from initial focus groups with Black, African American and Hispanic participants and families will provide perspectives on decision‐making, expectations, and post‐disclosure experiences. This study is anticipated to yield novel insights into patient and family preferences for receiving high stakes ADRD diagnostic information and to identify potential gaps in current support mechanisms.

**Conclusion:**

The results from this focus group investigation will contribute to a more comprehensive understanding of the information and support needs of culturally diverse families post‐ADRD diagnosis. Ultimately, this study seeks to inform culturally responsive care practices and will be disseminated to AD Research Centers and dementia experts, fostering the enhancement of best practices for biomarker disclosure and dementia‐related education.

